# Pharmacokinetic Assessment of Staphylococcal Phage K Following Parenteral and Intra-articular Administration in Rabbits

**DOI:** 10.3389/fphar.2022.840165

**Published:** 2022-05-20

**Authors:** Katherine M.C. Totten, Scott A. Cunningham, Naomi M. Gades, Athema Etzioni, Robin Patel

**Affiliations:** ^1^ Department of Molecular Pharmacology and Experimental Therapeutics, Mayo Clinic College of Medicine, Mayo Clinic, Rochester, MN, United States; ^2^ Division of Clinical Microbiology, Department of Laboratory Medicine and Pathology, Mayo Clinic, Rochester, MN, United States; ^3^ Department of Comparative Medicine, Mayo Clinic, Scottsdale, AZ, United States; ^4^ Department of Pathobiology, College of Veterinary Medicine, Tuskegee University, Tuskegee, AL, United States; ^5^ Division of Infectious Diseases, Department of Medicine, Mayo Clinic, Rochester, MN, United States

**Keywords:** phage, pharmacology, periprosthetic joint infection, staphylococci, pharmacokinetics

## Abstract

The therapeutic value of phage as an alternative to antibiotics for the treatment of bacterial infections is being considered in the wake of mounting antibiotic resistance. In this study, the pharmacokinetic properties of *Staphylococcus aureus* phage K following intravenous and intra-articular administration were investigated in a rabbit model. Using a traditional plaque assay and a novel quantitative PCR assay to measure phage levels in specimens over time, it was found that intra-articularly administered phage enters the systemic circulation; that phage may be detected in synovial fluid up to 24 h following the intra-articular, but not intravenous, administration; and that qPCR-based enumeration is generally more sensitive than plaque enumeration, with fair to moderate correlation between the two methods. Findings presented should inform the design of phage therapy experiments and therapeutic drug monitoring in preclinical and human phage studies.

## Introduction

As viruses of bacteria, bacteriophages have gained renewed attention as a potential treatment for antibiotic-resistant infections. Antibacterial phage application has attained a few successes, including its approval for foodborne prophylaxis as a biologic that is generally regarded as safe (GRAS) by the United States (US) Food and Drug Administration (FDA). There are also case reports and case series in which administration of compassionate use monophage or phage cocktails was associated with the resolution of human bacterial infections, including the 2016 Patterson case, in which a phage cocktail was used to treat *Acinetobacter baumannii* necrotizing pancreatitis following antibiotic failure (Schooley et al., 2017). Conversely, the literature attests to instances in which phage therapy has failed to clear bacterial infection (Sarker et al., 2016; Jennes et al., 2017; LaVergne et al., 2018; Jault et al., 2019), and there likely exist additional unreported negative data. Such unexplained variation is the result of a knowledge deficit of basic phage biology, including phage pharmacology.

Phage therapy may be advantageous in the context of periprosthetic joint infection (PJI), a serious complication of joint arthroplasty that occurs in 1–3% of people who have undergone joint replacement surgery (Tande and Patel, 2014). More than 2 million people underwent joint replacement surgery in the United States in 2019 (American Academy of Orthopaedic Surgeons, 2019b; a), and this number is expected to increase over time. PJI is treated with a combination of surgery and antibiotics, but infection resolution is not always attained (Tande and Patel, 2014). We recently reported successful treatment of *Klebsiella pneumoniae* PJI with intravenous (IV) phage KpJHΦ2 (Cano et al., 2020), while treatment of three subsequent cases of *S. aureus* and *Staphylococcus epidermidis* PJI with custom phage IV and intra-articular (IA) delivery failed to result in clinical improvement (unpublished). Potential reasons for failure include inadequate local phage concentrations reaching the site of infection, host neutralization, rapid elimination, and/or emergence of phage resistance. These mixed results stimulated an interest in defining the pharmacology of phage with a focus on IV and IA delivery given the interest in PJI.

Relatively few studies have evaluated small-molecule drug profiles in the joint space. The literature describing drug access to the joint space generally addresses small-molecule antibiotics and non-steroidal antiinflammatory drugs, corticosteroids, or biomolecular precursors administered for the treatment of infection and arthritis (Evans et al., 2014). Antibiotics (amphenicols, macrolides, fluoroquinolones, and β-lactams) and antiinflammatory drugs access the synovial compartment when administered systemically to both healthy (Depenbrock et al., 2017; Knych et al., 2017; Schwameis et al., 2017; Uney et al., 2017; Langston et al., 2019; Krueger et al., 2020) and unhealthy (defined as having one or more infected or inflamed joints) subjects (Porter et al., 1970; Thomas et al., 1975; Kennedy, 1976; Caruso et al., 1980; Kennedy, 1983; Hinderling et al., 1988; Kraml et al., 1988; Brown et al., 1991; Day et al., 1991; Scott et al., 2004; Rodrigues et al., 2010). An isotope clearance study in healthy and rheumatoid arthritis (RA) patients demonstrated a longer isotope half-life—defined as the length of time required for the decay of one-half of administered atoms—in healthy individuals and in clinically uninvolved knees in RA cases as compared with diseased knees. Joint perfusion was greater in RA cases—with higher values observed in diseased than uninvolved joints—than in healthy patients (Porter et al., 1970). Drugs for which detailed joint pharmacologic data are available are smaller than 1,000 Da, the apparent cutoff for passive drug diffusion through cellular membranes (Matsson and Kihlberg, 2017). In contrast, the genome of phage K—the most well-known lytic staphylococcal phage—is 54,000 Da (O'Flaherty et al., 2004). Concerning biologics, a majority of pharmacokinetic (PK) research on therapeutic biologics has been conducted using monoclonal antibodies (mAb), clinically available formulations which involve antibodies as large as 150,000 Da (Al-Kofahi, 2019); these have been demonstrated to access the synovial space following systemic administration (Choy et al., 2000). Thus, it is not anticipated that the phage size would preclude joint access, but this has not been empirically demonstrated.

In light of these reports which provide helpful, though limited, information about drug accessibility to the synovial compartment, the aim of this study was to understand the potential physicochemical constraints of phage delivery. Studies evaluating the pharmacokinetics of phage in the synovial compartment following IA and IV administration are thus needed to support clinical phage therapy studies.

## Materials and Methods

*Animal procedures.* Nineteen male and 20 female New Zealand white rabbits (2–4 kg, Envigo, Indianapolis, IN) aged 4–11 months were randomized into four groups: healthy joints, IA phage administration (9 animals); healthy joints, IV phage administration (12 animals); experimental osteoarthritis, IA phage administration (9 animals); and experimental osteoarthritis, IV phage administration (9 animals). Three additional male New Zealand white rabbits aged 1–4 years were studied as untreated controls. A sample size of six was calculated *a priori* to detect the differences of 0.17 log_10_ across two cohorts of three animals at a single time point post-phage administration at a power of 0.8. All animals were permitted a three-day acclimation period upon arrival, socially housed, and allowed access to food, water, and environmental stimulation *ad libitum* for the duration of the experiments. Experimental knee osteoarthritis was established according to Vinod et al (2018). Briefly, under ketamine (Zoetis, Parsippany, NJ), xylazine (Vet One, Boise, ID), acepromazine (Vet One), and isoflurane (Piramal Critical Care, Bethlehem, PA) anesthesia, the left suprapatellar region was shaved and disinfected with povidone-iodine, followed by injection of 4 mg monosodium iodoacetate (Millipore Sigma cat. no. 57858, Burlington, MA) in 0.25 ml sterile water into the joint space. The joint was extended and contracted five times, and experimental osteoarthritis was allowed to establish over 4 weeks. Buprenorphine-sustained release (0.18 mg/kg intramuscular, ZooPharm) was administered for post-procedural analgesia. This study was approved by the Mayo Clinic Institutional Animal Care and Use Committee (A00005772-21).

*Bacteriophage.* High-titer (2 × 10^12^ pfu/mL ± 1 × 10^1^ pfu/mL), purified phage K (ATCC 19685-B1) in phage buffer (100 mM NaCl, 10 mM MgCl Tris, pH 8.0) was prepared by TAILΦR Labs of Baylor College of Medicine (Houston, TX), as previously described (Green et al., 2017; Gibson et al., 2019; Terwilliger et al., 2021). Working stocks passed in-house sterility and endotoxin testing (8 EU/mL endotoxin C). Stocks were light-protected and stored at 4°C until administration. On study day 28, animals received 0.05 ml phage [10^11^ ± 10^1^ plaque forming units (pfu)] IA administered as above, or IV through an ear catheter with subsequent port flushing by saline or heparinized saline.

*Specimen collection.* Blood was collected from the central auricular vein or artery (depending on vessel patency) under ketamine, xylazine, acepromazine, and isoflurane anesthesia at 0, 0.25, 0.5, 1, 2, 4, 6, 8, and 24 h, following phage administration and placed into K_2_EDTA Microtainer tubes [Becton Dickinson (Rebai et al.) cat. no. 363706, Franklin Lakes, NJ]. Following sacrifice at 1, 8, or 24 h post-administration, three rabbits from each group underwent joint lavage of the ipsilateral knee with 1 ml saline (Baxter 0.9% sodium chloride irrigation cat. no. 2F7122, Deerfield, IL), followed by the aspiration of synovial lavage fluid for phage enumeration. Additionally, the heart, lung, kidney, liver, and spleen were collected from each animal and stored on ice in saline in separate Falcon tubes (Corning Inc. cat. no. 352098, Corning, NY) until phage enumeration studies were performed. Blood in K_2_EDTA Microtainer tubes was collected prior to and 24 h after IV phage administration and analyzed using a Sysmex XT-iV instrument by a comprehensive complete blood count with reticulocyte hemoglobin and differential validation in rabbits (IDEXX Bioanalytics cat. no 62216, Columbus, MO). Blood films were evaluated to confirm the analyzer results.

*Histopathology analysis*. The stifle joints, heart, lung, kidney, liver, and spleen sections were preserved at room temperature in 10% neutral-buffered formalin (cat. no. 316–155, Thomas Scientific, Swedesboro, NJ) for ≥72 h prior to paraffin embedding, sectioning, mounting, and hematoxylin and eosin (H&E) staining. The slides were reviewed by veterinary pathologist (NMG) masked to a treatment group via brightfield microscopy using an Olympus BX45 microscope. The North American Science Associates, Inc. (NAMSI) Comprehensive Histopathology Scoring System for Biomaterial Implants was used to score tissue sections (Upman and Muench, 2004). Joint sections were stained with methylene blue and basic fuchsin to visualize the cartilage structure and subchondral bone density, and blindly reviewed using the Mankin osteoarthritis scale.

*Phage enumeration.* Phage loads in collected specimens were enumerated by plaque assay and a novel quantitative real-time PCR (qPCR) assay. Prior to quantification by either method, tissue samples were processed as follows: one to three small pieces were cut from each organ, weighed, and placed into 1 ml saline. Then, each sample was piston homogenized (Cole-Parmer LabGEN 125 cat. no. EW-04727-04, Vernon Hills, IL) into a uniform slurry, which served as an input material for the plaque assay and qPCR. Blood and synovial lavage fluid did not undergo pre-processing.

For the plaque assay, three to five colonies of host bacterium (*S. aureus* MYC5) were grown to McFarland 0.5 visual turbidity equivalent in trypticase soy broth (TSB) (BD cat. no. 211768) at 37°C with shaking. Two hundred and fifty microliters of culture was added to 3 ml of cooled, molten 0.5% trypticase soy agar (TSA) (cat. no. LP0011, Oxoid Holdings Ltd., Basingstoke, England) supplemented with 10 mM MgSO_4_ and poured over solidified 1% TSA. Tissues were serially diluted ten-fold in 90 µl saline; 1 µl of each dilution was spotted onto the surface of the double layer agar. Plates were incubated at 37°C in room air overnight, with plaques enumerated the following day.

Phage was also enumerated using a qPCR assay. Eighty microliters of tissue homogenate were placed into Maxwell^®^ RSC Tissue DNA Kit (cat. no. AS1610, Promega, Madison, WI) cartridges for DNA extraction using the corresponding instrument according to the manufacturer’s instructions. DNA extraction from whole blood and synovial lavage fluid was carried out using the Maxwell^®^ RSC Whole Blood DNA Kit (cat. no. AS1520, Promega). Oligonucleotide sequences targeting a 110 base pair region of the phage K DNA polymerase I gene, *polA* (Genbank Accession KF766114, Bethesda, MD), were designed with LightCycler™ Probe Design Software 2.0 (Roche Molecular Systems, Pleasanton, CA) (Supplementary Table S1). qPCR master mix included 0.25 μM forward primer, 0.5 μM reverse primer, and 0.3 μM probe with 1X LightCycler TaqMan Master enzyme (Roche). Complete master mix of 15 μl was combined with 5 μl sample extract in 20 µl capillaries on a Roche LightCycler 2.0. Thermocycling conditions were as follows: 10 min denaturation at 95°C; 45 cycles of PCR 95°C for 10 s, 55°C for 20 s, and 72°C for 20 s in quantitative analysis mode; one cycle of 10 s at 40°C to cool the capillaries. The results were reported in copies (cp) per microliter, calculated by LightCycler regression analysis (Roche). An external standard line was created from gBlocks™ material, constructed based on the *polA* target sequence (Integrated DNA Technologies, Coralville, IA). Each point was tested in triplicate to create the quantification line. A single point calibrator and negative control were extracted in parallel with samples and included in each qPCR run.

Analytical sensitivity of the assays was measured in terms of limits of quantitation and detection. A stock solution of phage K (3.3E9 pfu/ml) was divided into aliquots. One aliquot was subjected to DNA extraction using the Maxwell^®^ RSC Viral Total Nucleic Acid Purification Kit (cat. no. AS1330) and was quantified using the QuantiFluor^®^ ONE dsDNA System on a Quantus™ Fluorometer. Three extracts were diluted ten-fold in saline, and subjected to qPCR testing with each reaction run in duplicate. The second aliquot was diluted ten-fold in saline and tested by plaque assay. The limit of quantitation (LoQ) of the qPCR assay was defined as the lowest concentration of DNA detected with a crossing threshold value in six of six samples, while the limit of detection (LoD) was defined as the lowest concentration of DNA detected in six of six samples with instrument extrapolation (Supplementary Table S2) (Armbruster and Pry, 2008). The LoQ and LoD of the plaque assay were considered as the phage concentration corresponding to a single plaque measured on the first ten-fold dilution. Analytical specificity was determined by *in silico* analysis. Sequences of the primers and probes were queried in NCBI Nucleotide BLAST (megablast), filtering out models and uncultured sequences, to identify potential cross-reactivity.

*PK modeling.* Phage titers in blood underwent noncompartmental analysis with pharmacokinetic parameters calculated per the enumeration method for healthy and osteoarthritic rabbits using the IV bolus functionality for IV administration and the extravascular functionality for IA administration in PKSolver (Zhang et al., 2010).

*Statistical analysis.* Differences in phage titers between treatment conditions, as well as the effect of phage administration on hematologic parameters, were assessed by the Wilcoxon rank sum test. Nonparametric correlation between qPCR and plaque assay enumeration was calculated using Spearman’s ρ. Ninety-five percent confidence intervals were computed for pharmacokinetic parameters across replicates. Statistical analysis was conducted by JMP 14.1.0 software (SAS Inc., Cary, NC). All tests were two-sided with *α* = 0.05; *p*-values ≤ 0.05 were considered statistically significant. Figures were prepared using GraphPad Prism 9.2.0 software (GraphPad Software, San Diego, CA).

## Results

### Phage K Pharmacokinetics in Healthy Rabbits

**FIGURE 1:**
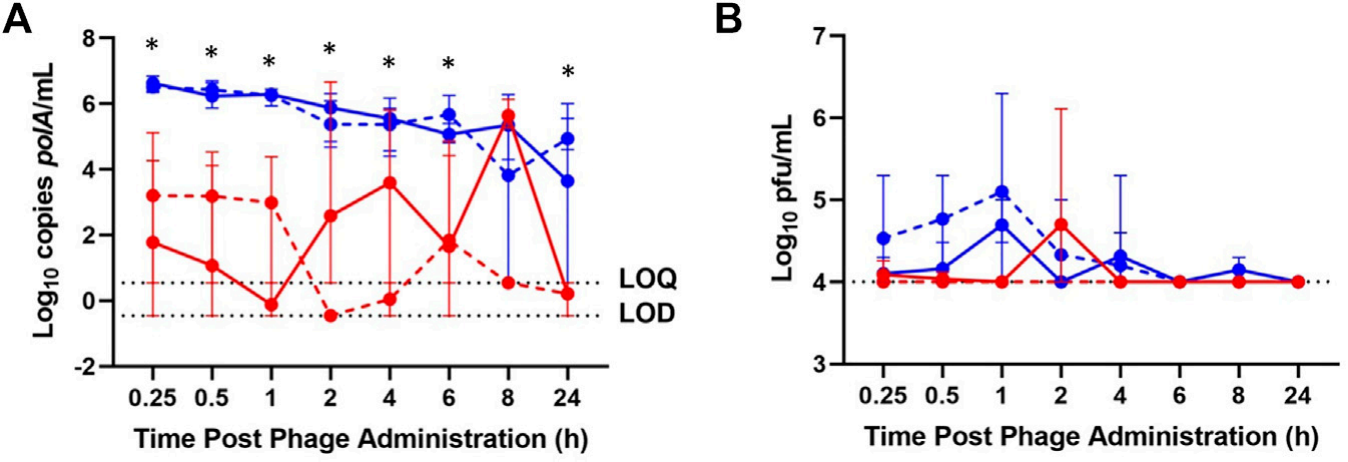
Phage K titers in blood of healthy (solid lines) and osteoarthritic (dashed lines) animals at various time points post-phage administration intra-articularly (red) or intravenously (blue) as measured by **(A)** qPCR and **(B)** plaque assay. Data points represent the average of three values, and error bars denote the range. *p ≤ 0.05 between intravenous and intra-articular concentrations (aggregated healthy and osteoarthritic conditions). *polA*/mL = cp/mL.

In healthy rabbits, between 3 and 7 log_10_ copies (cp)/mL of IV-administered phage was detected by qPCR in blood through 24 h, while generally 2–5 log_10_ cp/mL fewer IA-administered phage was detected in blood through 24 h (Figure 1A). IV-administered phage was detected by plaque assay in blood at 0.25, 0.5, 1, 4, and 8 h, attaining a maximum concentration of 4.7 log_10_ pfu/mL at 1 h post-administration; IA phage was detected in blood at 0.25, 0.5, and 2 h post-administration, also attaining a maximum concentration of 4.7 log_10_ pfu/mL at 2 h post-administration (Figure 1B).

**FIGURE 2:**
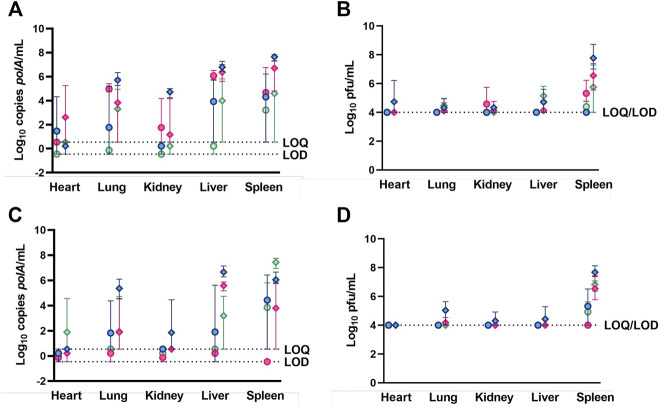
Phage K titers in tissues of healthy **(A,B)** and osteoarthritic **(C,D)** animals following sacrifice at 1-, 8-, and 24-h (blue, red, and green, respectively) post-phage administration intra-articularly (circles) or intravenously (diamonds) as measured by qPCR (a, c) or plaque assay (b, d). Data points represent the average of three values, and error bars denote the range. *polA*/mL = cp/mL.

IV-administered phage was detected by qPCR in the heart, lung, kidney, liver, and spleen between 1 and 24 h post-administration, generally attaining a maximum concentration at 1 h and declining through 24 h (Figure 2A). IA-administered phage was detected by qPCR in the heart, lung, kidney, liver, and spleen at all time points, excluding the heart and kidney at 24 h post-administration, and generally attained maximum concentrations at 8 h post-administration. Lower phage concentrations in log_10_ cp/mL in heart tissue were detected in healthy rabbits receiving IA versus IV phage at 24 h post-administration (*p* = 0.0469). IV-administered phage was detected by plaque assay in the heart, lung, kidney, liver, and spleen at 1 h; in the lung, kidney, liver, and spleen at 8 h; and in the lung, liver, and spleen at 24 h (Figure 2B). IA administered phage was only detected at 8 h in the kidney and spleen, and at 24 h in the spleen.

**FIGURE 3:**
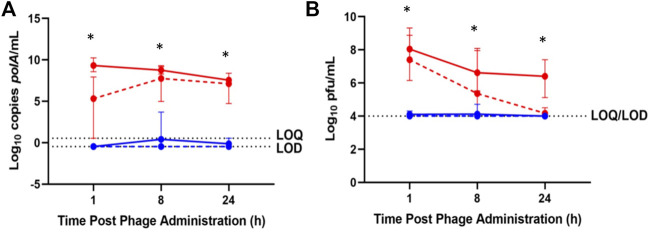
Phage K titers in synovial lavage fluid of healthy (solid lines) and osteoarthritic (dotted lines) animals following sacrifice at 1-, 8-, and 24-h post-phage administration intra-articularly (red) or intravenously (blue) as measured by **(A)** qPCR or **(B)** plaque assay. Data points represent the average of three values, and error bars denote the range. *p ≤ 0.05 between intravenous and intra-articular concentrations (aggregated healthy and osteoarthritic conditions). *polA*/mL = cp/mL.

Less than 1 log_10_ cp/mL IV-administered phage was detected by qPCR in synovial lavage fluid between 1 and 24 h, while IA-administered phage was detected above 7.5 log_10_ cp/mL throughout 24 h post-administration (Figure 3A). Similarly, low concentrations of IV-administered phage was detected by plaque assay in synovial lavage fluid at 1 and 8 h post-administration, with none detected at 24 h post-administration. In contrast, IA-administered phage was detected in synovial lavage fluid between 6 and 8 log_10_ pfu/mL through 24 h post-administration (Figure 3B). Finally, higher phage concentrations by log_10_ cp/mL and log_10_ pfu/mL were observed at 8 h post-administration in synovial lavage fluid with IA versus IV administered phage (*p* = 0.0219 and *p* = 0.0162, respectively).

### Phage K Pharmacokinetics in Osteoarthritic Rabbits

In the osteoarthritis model, 4 to 7 log_10_ cp/mL IV-administered phage was detected by qPCR in blood through 24 h post-administration, with IA phage detected by qPCR in blood at 0.25, 0.5, 1, 4, 6, 8, and 24 h post-administration, ranging from 3 to 6 log_10_ cp/mL less than that of samples following IV phage administration (Figure 1A). IV-administered phage was detected by plaque assay in blood through 6 h post-administration, attaining a maximum concentration of 5 log_10_ pfu/mL at 1 h, while no IA-administered phage was detected by plaque assay in blood through 24 h (Figure 1B).

IV-administered phage was detected in the heart, lung, kidney, liver, and spleen through 24 h post-administration (Figure 2C). The lung, kidney, and liver samples attained maximum concentrations at 1 h post-administration, while the heart and spleen attained maximum concentrations at 24 h post-administration. IA-administered phage was detected by qPCR in all tissues sampled through 24 h post-administration, except for the spleen, in which phage was detected through 8 h post-administration. IV-administered phage was detected by plaque assay at 1 h in the lung, kidney, liver, and spleen, at 8 h in the lung and spleen, and at 24 h in the spleen, generally attaining maximum concentrations at 1 h post-administration (Figure 2D). No IA-administered phage was detected by plaque assay in any tissue sampled except for the spleen between 1 and 24 h post-administration.

By both enumeration methods, rabbits receiving IV phage exhibited average synovial lavage fluid concentrations at the limit of detection through 24 h, while IA-administered phage attained concentrations between 1 and 9 log_10_ higher compared with IV administered phage through 24 h as measured by qPCR and plaque assays (Figure 3).

Blood concentrations over time were similar between healthy and osteoarthritic rabbits receiving IV and IA phage (Figure 1). Aggregate analyses showed a decrease in log_10_ cp/mL concentrations following IA- versus IV-administered phage at 0.25, 0.5, 1, 2, 4, 6, and 24 h post-administration (*p* = 0.0050, *p* = 0.0050, *p* = 0.0049, *p* = 0.0383, *p* = 0.0327, *p* = 0.0078 and *p* = 0.0099, respectively). Between healthy and osteoarthritic rabbits receiving IA or IV phage, osteoarthritic rabbits demonstrated a relative decrease in log_10_ cp/mL concentrations at 8 hours post-administration only (*p* = 0.0497). Aggregate analyses demonstrated a decrease in log_10_ pfu/mL concentrations of IA- versus IV-administered phage in blood at 1 h post-administration (*p* = 0.0095).

By both enumeration methods, phage concentrations were markedly higher in the spleen compared with other tissues sampled whether in healthy or osteoarthritic animals; high levels of phage was also detected in the lung and liver of both healthy and osteoarthritic rabbits by qPCR following IV administration (Figure 2). A decrease in log_10_ cp/mL in aggregated healthy and osteoarthritic rabbits receiving IA versus IV phage was observed in the kidney at 1 h post-administration (*p* = 0.0256); the lung (*p* = 0.0078 and *p* = 0.0396) and the liver (*p* = 0.0050 and *p* = 0.0256) between 1 and 24 h post-administration and the spleen (*p* = 0.0438) and the heart (*p* = 0.0225) at 24 h post-administration. Likewise, a decrease in log_10_ pfu/mL concentrations in rabbits receiving IA versus IV phage was observed in the spleen at 1, 8, and 24 h post-administration (*p* = 0.0043, *p* = 0.0036, and *p* = 0.0435, respectively).

IV-administered phage concentrations in synovial lavage fluid followed similar trends in healthy and osteoarthritic animals as assessed by both enumeration methods; similarly, IA-administered phage concentrations in synovial lavage fluid were similar between healthy and osteoarthritic rabbits with both enumeration methods (Figure 3). Increased phage concentrations in synovial lavage fluid by log_10_ cp/mL (*p* = 0.0028, *p* = 0.0010, and *p* = 0.0037) and log_10_ pfu/mL (*p* = 0.0037, *p* = 0.0206, and *p* = 0.0284) in healthy and osteoarthritic rabbits were observed following IA- versus IV-administered phage at 1, 8, and 24 h post-administration.

No polymorphonuclear cells, lymphocytes, plasma cells, macrophages, giant cells, or necrosis—collectively indicative of inflammation—as reflected by NAMSI Comprehensive Histopathology Scoring System for Biomaterial Implants scores, were observed in the heart, lung, kidney, liver, or spleen tissue of any phage-treated or control animals. Peribronchiolar lymph node hyperplasia was noted in lung sections of 10 (three healthy and seven osteoarthritic) phage-treated animals. Papillary proliferation of the bronchial epithelium and inflammatory cell extravasation of lung parenchyma were each noted in one healthy, phage-treated rabbit. No indications of osteoarthritis, such as surface irregularities, bone clefts or other structural abnormalities, and staining or tidemark disintegrity were observed in stifle joint sections across treatment and control groups.

Hematology studies showed similar values in blood sampled before and 24 h after IV phage administration (Table 1) and were generally consistent with reference ranges for New Zealand white rabbits (Hewitt et al., 1989; Ozkan et al., 2012; Moore et al., 2015). Heterophils (equivalent to neutrophils) doubled 24 h post-phage administration, while percent lymphocytes decreased over this time frame, albeit remaining within normal range.

**TABLE 1:** Hematology values from samples collected before and 24 h after intravenous phage administration in three rabbits. NA = not available.

	Pre-phage administration			24 h post-phage administration		
Analyte	Rabbit			Rabbit		
	1	2	3	1	2	3
Leukocyte (K/µL)	5.7	7.5	5.5	7	5.5	7.2
Red blood cell (M/µL)	5.11	5.36	5.76	5.88	6.07	6.08
Hemoglobin (g/dl)	11	11.6	11.6	11.7	12.5	12.5
Hematocrit (%)	36.3	38.6	37.9	38.6	39.8	39.7
Mean corpuscular volume (fL)	71	72	66	66	66	65
Mean corpuscular hemoglobin (pg)	21.5	21.6	20.1	19.9	20.6	20.6
Mean corpuscular hemoglobin concentration (g/dl)	30.3	30.1	30.6	30.3	31.4	31.5
Platelets (K/µL)	328	158	314	328	213	185
Reticulocyte (K/µL)	206	228	157	153	134	142
Nucleated RBC (/100 WBC)	2	1	2	0	2	0
Polychromasia	Slight	Moderate	Slight	Slight	Slight	Not seen
Anisocytosis	Slight	Slight	Slight	Slight	Slight	Not seen
Poikilocytosis	Slight	Slight	Not seen	Slight	Slight	Not seen
Heinz bodies	Not seen	Not seen	Not seen	Not seen	Not seen	Not seen
Neutrophil (/µL)	1,596	2,475	2,585	4,340	1,980	3,888
Band (%)	0	0	0	0	0	0
Lymphocytes (/µL)	3,534	4,575	2,200	2,240	2,860	2,880
Monocytes (/µL)	228	225	385	350	440	144
Eosinophils (/µL)	342	225	330	70	220	288

Pharmacokinetic analysis yielded comparable parameter values by both assays between healthy and OA conditions whether by IV or IA delivery routes (Tables 2 and 3). Parameters taking into account the terminal elimination phase could not be calculated for all conditions because of non-diminishing concentrations observed at final time points.

**TABLE 2:** Pharmacokinetic parameters of intravenously administered phage. λz = terminal elimination rate constant; t_1/2_ = half-life; T_max_ = time at which drug attains maximum concentration; C_max_ = maximum concentration; C_0_ = initial blood concentration; AUC_0-t_ = area under the curve from time 0 to last measurable concentration; AUC_0-∞_ = area under the curve extrapolated to infinity; AUMC = area under the moment curve; MRT = mean residence time; Vz = terminal elimination phase volume of distribution; Cl = clearance; Vss = steady state volume of distribution; * = could not be calculated.

Parameter	Plaque assay [mean (95% CI)]			qPCR assay [mean (95% CI)]		
	Units	Healthy	Osteoarthritic	Units	Healthy	Osteoarthritic
λz	1/h	2.571E-02 (−2.74E-2—9.88E-2)	*	1/h	2.770E-01 (−4.88E-1—1.04E0)	1.350E-01 (−1.92E-2—2.89E-1)
t_1/2_	h	1.979E+01 (−1.52E1—5.48E1)	*	h	1.530E+01 (−4.04E1—7.09E1)	6.040E+00 (−1.54E0—1.36E1)
T_max_	h	1.000E+00 (1E1—1E1)	1.167E+00 (−7.31E-1—3.06E0)	h	5.830E-01 (−3.65E-1—1.53E0)	3.330E-01 (−2.52E-2—6.92E-1)
C_max_	pfu/mL	5.667E+04 (−3.73E4—1.51E5)	7.649E+05 (−1.89E6—3.42E6)	cp/mL	4.840E+06 (−8.65E5—1.06E7)	3.940E+06 (3.07E6—4.81E6)
C_0_	pfu/mL	1.332E+04 (-9.57E2—2.76E4)	0.000E+00 (0.00E0—0.00E0)	cp/mL	2.520E+07 (-6.44E7—1.15E8)	5.200E+06 (-8.77E5—1.13E7)
AUC_0-t_	pfu/mL*h	3.518E+05 (1.86E5—5.18E5)	8.740E+05 (−1.14E6—2.89E6)	cp/mL*h	2.630E+07 (6.16E6—4.64E7)	1.040E+07 (−9.33E5—2.16E7)
AUC_0-∞_	pfu/mL*h	6.757E+05 (2.60E5—1.09E6)	*	cp/mL*h	4.610E+07 (−2.34E7—1.16E8)	1.210E+07 (−4.74E6—2.9E7)
AUC_0-t/0-∞_		5.792E-01 (9.17E-2—1.07E0)	*		7.450E-01 (−3.43E-1—1.83E0)	8.950E-01 (5.80E-1—1.21E0)
AUMC	pfu/mL*h^2^	1.885E+07 (−2.23E7—6.00E7)	*	cp/mL*h^2^	1.820E+09 (−5.37E9—9.02E9)	1.260E+08 (−2.42E8—4.93E8)
MRT	h	2.773E+01 (-1.61E1—7.16E1)	*	h	2.550E+01 (-6.22E1—1.13E2)	8.050E+00 (-7.90E0—2.40E1)
Vz	pfu/(pfu/ml)	4.208E+06 (−6.67E5—9.08E6)	*	cp/(cp/mL)	1.190E+05 (−2.13E6—4.51E6)	2.670E+06 (1.49E6—3.84E6)
Cl	pfu/(pfu/ml)/h	1.483E+05 (5.70E4—2.40E5)	*	cp/(cp/mL)/h	9.560E+04 (−2.14E4—2.13E5)	3.740E+05 (−2.00E5—9.48E5)
Vss	pfu/(pfu/ml)	4.089E+06 (1.16E5—8.06E6)	*	cp/(cp/mL)	1.430E+06 (-2.03E6—4.88E6)	2.200E+06 (7.39E5—3.66E6)

**TABLE 3:** Pharmacokinetic parameters of intra-articularly administered phage. λz = terminal elimination rate constant; t_1/2_ = half-life; T_max_ = time at which drug attains maximum concentration; C_max_ = maximum concentration; AUC_0-t_ = area under the curve from time 0 to last measurable concentration; AUMC_0-**∞**_ = area under the moment curve extrapolated to infinity; MRT_0-∞_ = mean residence time extrapolated to infinity; Vz/F_obs_ = apparent volume of distribution; * = could not be calculated.

Parameter	Plaque assay (mean (95% CI)]			qPCR assay (mean (95% CI)]		
	Units	Healthy	Osteoarthritic	Units	Healthy	Osteoarthritic
λz	1/h	*	*	1/h	*	2.263E-01 (−9.45E-1—1.40E0)
t_1/2_	h	*	*	h	*	3.674E+00 (−1.54E1—2.27E1)
T_max_	h	8.333E-01 (−1.68E0—3.34E0)	2.500E-01 (2.50E-1—2.50E-1)	h	6.000E+00 (−2.61E0—1.46E1)	3.333E-01 (−2.52E-2—6.92E-1)
C_max_	pfu/mL	4.388E+05 (−1.39E6—2.27E6)	1.000E+03 (1.00E3—1.00E3)	cp/mL	2.055E+06 (−3.62E6—7.73E6)	7.285E+04 (−9.46E4—2.40E5)
AUC_0-t_	pfu/mL*h	8.789E+05 (−1.87E6—3.63E6)	2.388E+05 (−6.30E6—2.37E7)	cp/mL*h	8.705E+06 (−6.30E6—2.37E7)	6.138E+04 (−8.74E4—2.10E5)
AUC_0-∞_	pfu/mL*h	*	*	cp/mL*h	*	5.990E+04 (−7.01E5—8.20E5)
AUC_0-t_/_0-∞_		*	*		*	9.764E-01 (6.78E-1—1.28E0)
AUMC_0-∞_	pfu/mL*h^2^	*	*	cp/mL*h^2^	*	2.471E+05 (−2.89E6—3.38E6)
MRT_0-∞_	h	*	*	h	*	6.350E+00 (−2.19E1—3.47E1)
Vz/F_obs_	pfu/(pfu/ml)	*	*	cp/(cp/mL)	*	2.390E+11 (−2.80E12—3.27E12)

The LoD and LoQ of the plaque assay was 10,000 pfu/ml. Phage-untreated tissues did not contribute to any signal in the *polA* qPCR (Supplementary Figure S1). The LoQ of the qPCR assay was 3,500 cp/mL and the LoD was 350 cp/mL (Supplementary Table S2), thus reflecting an increased sensitivity compared with the plaque assay. *In silico* analysis of qPCR assay specificity by a NCBI Nucleotide BLAST search returned 89 entries corresponding to staphylococcal phage for the forward primer and probe, and 68 entries corresponding to staphylococcal phage for the reverse primer and probe. Further analysis revealed viral sequence similarity to the members of the subfamily *Twortvirinae* (order *Caudovirales*, family Herelleviridae), which includes the genus *Kayvirus* to which phage K belongs.

Spearman’s ρ analysis of correlation between enumeration results by plaque assay versus qPCR ranged from 0.500 to 0.7314 for blood, tissue, synovial lavage fluid, and aggregate specimens (*p* < 0.0001, Supplementary Figure S2).

## Discussion

This study is among few investigations of native phage pharmacokinetics to date (Gill et al., 2006; Chow et al., 2020; Lin et al., 2020; Dhungana et al., 2021); this is the second publication to consider such properties of staphylococcal phage K, and the first to evaluate phage residence time and distribution in the synovial compartment. Toward the aim of describing physicochemical characteristics of intravenously and intra-articularly administered phage, the data suggest that intravenously administered phage may access the synovial compartment and intra-articularly administered phage may access the central compartment.

Although phage was detected in blood following both IV and IA administrations, significantly higher concentrations (cp/mL) were attained from IV versus IA administration through 6 h post-administration. For more controlled circulation time and tissue localization of intravenously administered phages, numerous encapsulation strategies have been and are currently being pursued, including liposomes and hydrogel formulations (Loh et al., 2021). These approaches have the added benefit of concealing phages immune cells to prevent the emergence of phage-neutralizing antibodies. Meanwhile, phage was detected in synovial lavage fluid at all time points following the IA administration in healthy and osteoarthritic rabbits, except at 24 h in osteoarthritic rabbits; significantly lower concentrations (cp/mL and pfu/mL) were attained at this site following IV administration. Sustained phage concentrations were observed in the lung, liver, and spleen of healthy and osteoarthritic, IV phage-treated rabbits, as well as healthy and osteoarthritic, IA phage-treated rabbits, though to a reduced degree.

Disparities in phage concentrations by administration route and assay type were observed. Variability in phage concentrations across specimens following IA compared with IV administration was expected due to technical challenges concerning the access of the stifle joint. Even in comparatively large human joints, as many as half of aspirations sample adjacent material rather than intended synovial fluid (Jones et al., 1993; Jackson et al., 2002; Evans et al., 2014). With few exceptions, qPCR detected greater viral loads than plaque assay. This is not surprising, given that PCR detects target nucleic acid of viable and non-viable phages, while the plaque assay detects viable, infectious phage only. Further, contributing to underestimated phage loads measured by plaque assay is that multiple phages might be captured within a single plaque (conventionally equivalent to one phage) as newly synthesized viruses are radially disseminated. This study did not investigate whether differential phage loads detected by the plaque assay and qPCR were due to phage degradation or inactivation in transit (resulting in a qPCR signal but no plaques), detection of circulating non-viable phage by qPCR, or some combination of the two. Correlational analyses between viral loads detected by each method yielded a Spearman’s ρ ranging from 0.5000 to 0.7314. Spearman’s ρ is a rank-based measure of association between two variables on a scale from -1 to 1, in which a correlation coefficient (ρ) of -1 corresponds to a perfect, negative monotonic relationship, and 1 corresponds to a perfect, positive monotonic relationship. The coefficients describing the relationship between plaque assay and qPCR assay outputs by the specimen type are assigned fair (tissue, blood, and aggregated specimens) to moderate (synovial lavage fluid) interpretations of correlation according to one algorithm (Chan, 2003; Akoglu, 2018).

Hematologic parameters were generally similar between blood sampled pre-phage and 24 h post-phage and were consistent with published reference ranges (Hewitt et al., 1989; Ozkan et al., 2012; Moore et al., 2015), while median platelet counts fell below the normal range in one pre-phage sample and two post-phage samples (Table 1). This may be due to the clotting noted in five of six blood samples and is therefore likely a processing artifact. Changes originating in the bone marrow arise three to four days after exposure; thus, this study is unlikely to have assessed the full immunogenic potential, if any, of phage K.

Ubiquitously negative scores on the NAMSI Comprehensive Histopathology Scoring System for Biomaterial Implants across tissue types and administration routes suggest that low-endotoxin phage preparations do not cause host tissue irritation. Peribronchiolar lymph node hyperplasia was noted in lung sections of phage-treated animals may have been associated with antigenic stimulation (Sirajuddin et al., 2016). Because this observation was noted in tissue sections of osteoarthritic, phage-treated rabbits with twice the frequency of healthy, phage-treated rabbits, and not observed in untreated controls, it is unclear whether monosodium iodoacetate acts as such a stimulator or whether this is an incidental finding due to an unknown inflammatory event given the small number of animals studied. As inflammatory cell migration is observed at least three days after an immunogenic event, this finding is not expected to be the result of phage K administration. No correlation between monosodium iodoacetate and pulmonary inflammation has been reported. Papillary proliferation of the bronchial epithelium and inflammatory cell extravasation were also observed in lung sections of one healthy, phage-treated rabbit each. Similarly, while pulmonary epithelial proliferation has been observed in severe asthma (Cohen et al., 2007) and infection (Mock et al., 2014), and, together, with observed inflammatory cell migration may indicate local injury, cell migration and proliferation are here expected to be the result of an unknown inflammatory event unrelated to phage administration.

Significant differences in pharmacokinetic parameter values for either assay were not detected between healthy and OA conditions within IV or IA delivery routes (Tables 2 and 3). Parameters based on the terminal elimination phase could not be calculated for all conditions because of non-diminishing concentrations observed at final time points, which may be explained by low sensitivity of the plaque assay or a narrow sampling time frame that may not have captured phage efflux from the central compartment.

The primary limitation of this study is the single-dose, single-phage design with sample collection taking place over a short time period. Given the apparent safety of phage therapy, many published cases employ a repeated dosing strategy to maximize antibacterial potential, such that results of this single-dose study may not be directly comparable. Another limitation is a failure to confirm the histopathologic evidence of osteoarthritis among rabbits receiving monosodium iodoacetate. Although disease induction was undertaken as previously described (Vinod et al., 2018; Rebai et al., 2020), the absence of arthritic changes observed mitigates the significance of results in the osteoarthritis group. Furthermore, while phage K is the best-studied staphylococcal phage, the observed trends in biodistribution and residence time may not generalize to other phages, even other staphylococcal phages. Enumerating phage in whole blood rather than plasma or serum—the standard specimens for pharmacokinetic profiling—due to small blood volumes sampled and uncertainty surrounding the blood fraction in which phage is sequestered—may hinder comparison with other pharmacokinetic studies. Another study limitation is the small sample size and pre-analytic errors in the blood sampling that resulted in platelet clumping and pseudothrombocytopenia, with additional analytes potentially also being impacted. Finally, the design of the qPCR assay affords the remote possibility of detecting background staphylococcal phage. However, as obligate parasites of bacteria, phages are not anticipated to reside in niches lacking a host; as animals were not infected and procedures were performed with clean technique, the presence, and subsequent detection, of off-target phages is unlikely. Furthermore, naive rabbit specimens did not contribute a signal to the assay (Supplementary Figure S1); rather, the described qPCR assay which detects the conserved (Nasko et al., 2018; Benler et al., 2021) *polA* phage gene may provide a tool to develop a standardized assay for monitoring phage concentrations in samples following administration of other *Twortvirinae*.

Future studies should address the impact of repeated administration on phage pharmacokinetics and whether development of neutralizing antibodies differs by the route of administration, in addition to the impact of bacterial infection and phage encapsulation on biodistribution and residence time. It should be noted that phage-bacterial interactions measured in the laboratory may diverge from those in uncontrolled environments (Hussain et al., 2021; Meaden and Fineran, 2021). While phage doses for clinical use are as-yet-undefined, the data presented herein may facilitate the design of treatment plans to achieve antibacterial effects at particular sites of infection.

Collectively, the results of this study demonstrate widespread phage distribution over a 24 h period following IV or IA administration, albeit with low viral loads observed in synovial lavage fluid following IV administration and in blood following IA administration. Joint inflammation modeled by unilateral osteoarthritis induction of the knee led to decreased phage concentrations in synovial lavage fluid across all time points sampled compared with healthy, phage-treated rabbits, which may suggest the possibility of a longer dosing interval for IA phage administration in the setting of PJI, septic arthritis, and similar conditions. Phage enumeration by the qPCR and plaque assays demonstrated fair to moderate correlation by Spearman’s ρ, although qPCR tended to yield higher phage loads. Hematologic studies did not reveal abnormalities post-phage administration. Further studies are needed to understand the generalizability of these results to other model species, other phages, repeated dosing designs, and in the context of infection.

## Data Availability

A publicly available dataset was utilized in this study. This data can be found here: Genbank Accession Number KF766114, https://www.ncbi.nlm.nih.gov/nuccore/KF766114.
