# Corrigendum: Pharmacokinetic assessment of staphylococcal phage K following parenteral and intra-articular administration in rabbits

**DOI:** 10.3389/fphar.2022.1005123

**Published:** 2022-09-02

**Authors:** Katherine M. C. Totten, Scott A. Cunningham, Naomi M. Gades, Athema Etzioni, Robin Patel

**Affiliations:** ^1^ Department of Molecular Pharmacology and Experimental Therapeutics, Mayo Clinic College of Medicine, Mayo Clinic, Rochester, MN, United States; ^2^ Division of Clinical Microbiology, Department of Laboratory Medicine and Pathology, Mayo Clinic, Rochester, MN, United States; ^3^ Department of Comparative Medicine, Mayo Clinic, Scottsdale, AZ, United States; ^4^ Department of Pathobiology, College of Veterinary Medicine, Tuskegee University, Tuskegee, AL, United States; ^5^ Division of Infectious Diseases, Department of Medicine, Mayo Clinic, Rochester, MN, United States

**Keywords:** phage, pharmacology, periprosthetic joint infection, staphylococci, pharmacokinetics

In the published article, there was an error. There was a typographical error included in the American Type Culture Collection (ATCC) reference number of phage K.

A correction has been made to **Materials and Methods**, *Bacteriophage*. This sentence previously stated:

“High-titer (2 × 10^12^ pfu/ml ± 1 × 10^1^ pfu/ml) purified phage K (ATCC 19695-B1) in phage buffer (100 mM NaCl, 10 mM MgCl Tris, pH 8.0) was prepared by TAILΦR Labs of Baylor College of Medicine (Houston, TX), as previously described (Green et al., 2017; Gibson et al., 2019; Terwilliger et al., 2021). Working stocks passed in-house sterility and endotoxin testing (8 EU/mL endotoxin C). Stocks were light-protected and stored at 4°C until administration. On study day 28, animals received 0.05 ml phage [10^11^ ± 10^1^ plaque forming units (pfu)] IA administered as above, or IV through an ear catheter with subsequent port flushing by saline or heparinized saline.”

The corrected sentence appears below:

“High-titer (2 × 10^12^ pfu/mL ± 1 × 10^1^ pfu/mL) purified phage K (ATCC 19685-B1) in phage buffer (100 mM NaCl, 10 mM MgCl Tris, pH 8.0) was prepared by TAILΦR Labs of Baylor College of Medicine (Houston, TX), as previously described (Green et al., 2017; Gibson et al., 2019; Terwilliger et al., 2021). Working stocks passed in-house sterility and endotoxin testing (8 EU/mL endotoxin C). Stocks were light-protected and stored at 4°C until administration. On study day 28, animals received 0.05 ml phage [10^11^ ± 10^1^ plaque forming units (pfu)] IA administered as above, or IV through an ear catheter with subsequent port flushing by saline or heparinized saline.”

The authors apologize for this error and state that this does not change the scientific conclusions of the article in any way. The original article has been updated.

