# Supplementary material for: Pharmacokinetic Assessment of Staphylococcal Phage K Following Parenteral and Intra-articular Administration in Rabbits
Source: Front Pharmacol. 2022 May 20;13:840165. doi: 10.3389/fphar.2022.840165 (PMC9163985; doi:10.3389/fphar.2022.840165)
Supplement: Supplementary file 1 [file DataSheet1.docx]

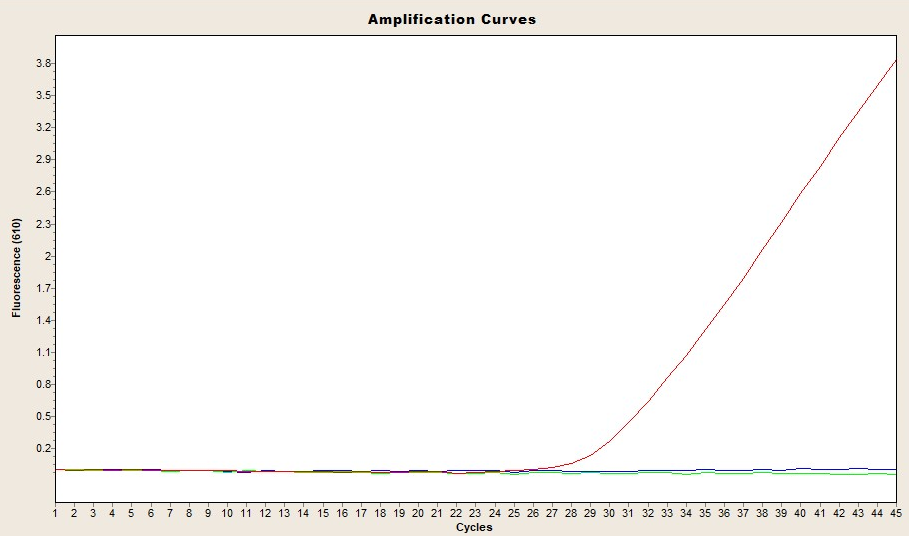

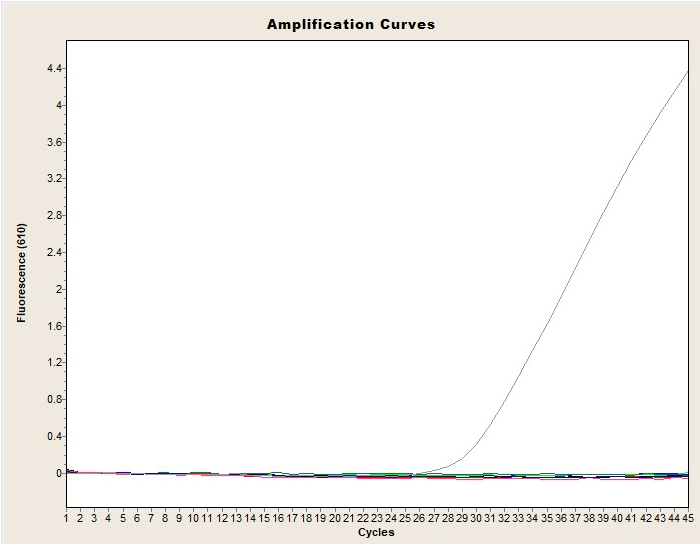


| Color | Pos | Name | Cycle threshold |
| --- | --- | --- | --- |
|  | 1 | Negative control | ND |
|  | 2 | Heart | ND |
|  | 3 | Liver | ND |
|  | 4 | Spleen | ND |
|  | 5 | Lung | ND |
|  | 6 | Kidney | ND |
|  | 7 | Synovial fluid | ND |
|  | 8 | Positive control | 28.41 |

| Color | Pos | Name | Cycle threshold |
| --- | --- | --- | --- |
|  | 1 | Negative control | ND |
|  | 2 | Whole blood | ND |
|  | 3 | Positive control | 28.27 |

Figure S1. Rabbit blood, synovial lavage fluid, and tissues do not contribute a signal to the *polA* qPCR assay.


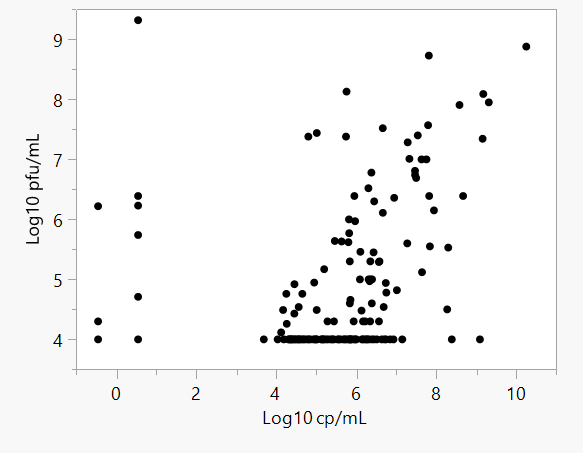

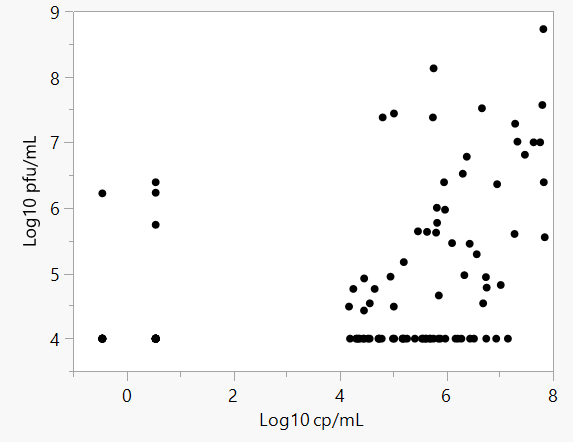

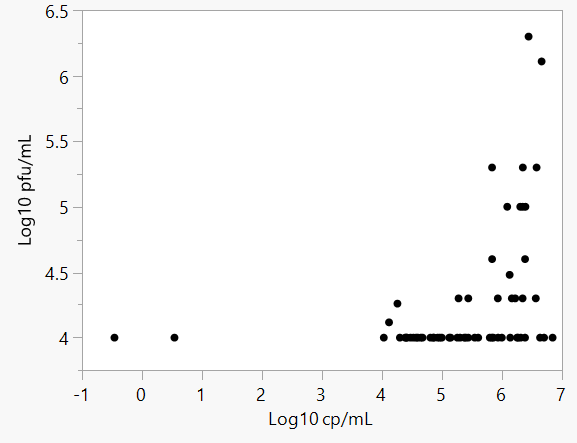

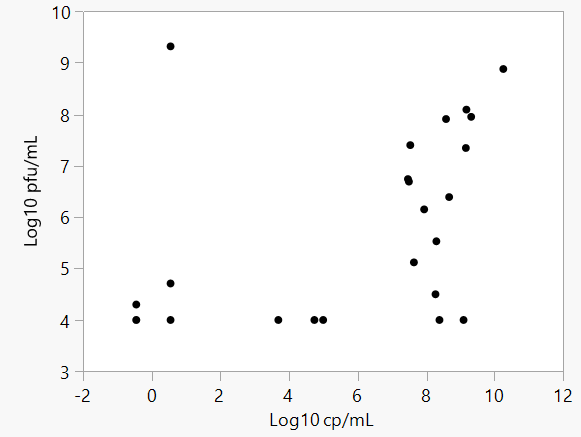


**a**

**b**

**c**

**d**

Figure S2. Correlation of phage enumeration results by plaque assay (log_10_ pfu/mL) and *polA* assay (log_10_ cp/mL) in tissue (a), blood (b), synovial lavage fluid (c), and aggregate specimens (d).

Table S1. Phage K *polA* qPCR primers (Integrated DNA Technologies, Coralville, IA).

| **Oligo Name** | **Sequence (5’–3’)^a^** |
| --- | --- |
| polA110F | CTAGAATTGAATCAAATGGTTTATATTGTG |
| polA110R | CCAGTGCTCTCTCATTGTAG |
| polA110TqM | 5TexRd-XN-GTCATACGAGTCTGAGATGGCTAAGAAC-3IAbRQSp |

^a^TEXRd-XB, Texas Red®; IAbRQSp, Iowa Black quencher

Table S2. Analytical sensitivity of *polA* qPCR assay. The limit of quantitation was defined as the lowest value where the assay produced a positive cycle threshold result in 6 of 6 replicates (3.5E0 genome copies/μL), and the limit of detection was defined as the lowest value where the assay produced a positive result in 6 of 6 replicates (3.5E-1 genome copies/μL). ND=not detected.

| **Genome copies/μL** | **Cycle Threshold Value** | | | | | | **Mean Cycle Threshold Value** |
| --- | --- | --- | --- | --- | --- | --- | --- |
| 3.50E+07 | 10.88 | 11.20 | 11.20 | 10.79 | 11.03 | 11.05 | 11.03 |
| 3.50E+06 | 13.63 | 14.09 | 14.06 | 13.72 | 14.32 | 14.08 | 13.98 |
| 3.50E+05 | 17.24 | 17.61 | 17.49 | 17.35 | 17.44 | 17.36 | 17.42 |
| 3.50E+04 | 20.85 | 21.01 | 21.03 | 20.83 | 21.08 | 21.00 | 20.97 |
| 3.50E+03 | 24.16 | 24.63 | 24.20 | 24.17 | 24.52 | 24.28 | 24.33 |
| 3.50E+02 | 27.70 | 27.91 | 27.90 | 27.71 | 27.73 | 27.87 | 27.80 |
| 3.50E+01 | 31.17 | 31.19 | 31.40 | 31.01 | 31.24 | 31.31 | 31.22 |
| 3.50E+00 | 34.50 | 34.25 | 34.42 | 34.47 | 34.84 | 34.38 | 34.48 |
| 3.50E-01 | >40.0 | 39.56 | >40.0 | >40.0 | 36.88 | 33.41 | 36.62 |
| 3.50E-02 | ND | ND | >40.0 | ND | ND | >40.0 |  |
| 3.50E-03 | ND | ND | ND | ND | ND | ND |  |
